# Cardiovascular Implications of Fatal Outcomes of Patients With Coronavirus Disease 2019 (COVID-19)

**DOI:** 10.1001/jamacardio.2020.1017

**Published:** 2020-03-27

**Authors:** Tao Guo, Yongzhen Fan, Ming Chen, Xiaoyan Wu, Lin Zhang, Tao He, Hairong Wang, Jing Wan, Xinghuan Wang, Zhibing Lu

**Affiliations:** 1 Department of Cardiology, Zhongnan Hospital of Wuhan University, Wuhan, China; 2 Department of Urology, Zhongnan Hospital of Wuhan University, Wuhan, China

## Abstract

**Importance:**

Increasing numbers of confirmed cases and mortality rates of coronavirus disease 2019 (COVID-19) are occurring in several countries and continents. Information regarding the impact of cardiovascular complication on fatal outcome is scarce.

**Objective:**

To evaluate the association of underlying cardiovascular disease (CVD) and myocardial injury with fatal outcomes in patients with COVID-19.

**Design, Setting, and Participants:**

This retrospective single-center case series analyzed patients with COVID-19 at the Seventh Hospital of Wuhan City, China, from January 23, 2020, to February 23, 2020. Analysis began February 25, 2020.

**Main Outcomes and Measures:**

Demographic data, laboratory findings, comorbidities, and treatments were collected and analyzed in patients with and without elevation of troponin T (TnT) levels.

**Results:**

Among 187 patients with confirmed COVID-19, 144 patients (77%) were discharged and 43 patients (23%) died. The mean (SD) age was 58.50 (14.66) years. Overall, 66 (35.3%) had underlying CVD including hypertension, coronary heart disease, and cardiomyopathy, and 52 (27.8%) exhibited myocardial injury as indicated by elevated TnT levels. The mortality during hospitalization was 7.62% (8 of 105) for patients without underlying CVD and normal TnT levels, 13.33% (4 of 30) for those with underlying CVD and normal TnT levels, 37.50% (6 of 16) for those without underlying CVD but elevated TnT levels, and 69.44% (25 of 36) for those with underlying CVD and elevated TnTs. Patients with underlying CVD were more likely to exhibit elevation of TnT levels compared with the patients without CVD (36 [54.5%] vs 16 [13.2%]). Plasma TnT levels demonstrated a high and significantly positive linear correlation with plasma high-sensitivity C-reactive protein levels (β = 0.530, *P* < .001) and N-terminal pro–brain natriuretic peptide (NT-proBNP) levels (β = 0.613, *P* < .001). Plasma TnT and NT-proBNP levels during hospitalization (median [interquartile range (IQR)], 0.307 [0.094-0.600]; 1902.00 [728.35-8100.00]) and impending death (median [IQR], 0.141 [0.058-0.860]; 5375 [1179.50-25695.25]) increased significantly compared with admission values (median [IQR], 0.0355 [0.015-0.102]; 796.90 [401.93-1742.25]) in patients who died (*P* = .001; *P* < .001), while no significant dynamic changes of TnT (median [IQR], 0.010 [0.007-0.019]; 0.013 [0.007-0.022]; 0.011 [0.007-0.016]) and NT-proBNP (median [IQR], 352.20 [174.70-636.70]; 433.80 [155.80-1272.60]; 145.40 [63.4-526.50]) was observed in survivors (*P* = .96; *P* = .16). During hospitalization, patients with elevated TnT levels had more frequent malignant arrhythmias, and the use of glucocorticoid therapy (37 [71.2%] vs 69 [51.1%]) and mechanical ventilation (31 [59.6%] vs 14 [10.4%]) were higher compared with patients with normal TnT levels. The mortality rates of patients with and without use of angiotensin-converting enzyme inhibitors/angiotensin receptor blockers was 36.8% (7 of 19) and 21.4% (36 of 168) (*P* = .13).

**Conclusions and Relevance:**

Myocardial injury is significantly associated with fatal outcome of COVID-19, while the prognosis of patients with underlying CVD but without myocardial injury is relatively favorable. Myocardial injury is associated with cardiac dysfunction and arrhythmias. Inflammation may be a potential mechanism for myocardial injury. Aggressive treatment may be considered for patients at high risk of myocardial injury.

## Key Points

**Question:**

What is the impact of underlying cardiovascular disease (CVD) and myocardial injury on fatal outcomes in patients with coronavirus disease 2019 (COVID-19)?

**Findings:**

In this case series study of 187 patients with COVID-19, 27.8% of patients had myocardial injury, which resulted in cardiac dysfunction and arrhythmias. Myocardial injury has a significant association with fatal outcome of COVID-19, while the prognosis of patients with underlying CVD but without myocardial injury were relatively favorable.

**Meaning:**

It is reasonable to triage patients with COVID-19 according to the presence of underlying CVD and evidence of myocardial injury for prioritized treatment and even more aggressive strategies.

## Teaser

This case series study evaluates the association of underlying cardiovascular disease and myocardial injury on fatal outcomes in patients with coronavirus disease 2019 (COVID-19).

## Introduction

Coronavirus disease 2019 (COVID-19) is a newly recognized infectious disease that has spread rapidly throughout Wuhan, Hubei, China, to other provinces in China and several countries around the world. The number of fatalities owing to COVID-19 is escalating. Previous studies have described the general clinical characteristics and epidemiological findings of patients with COVID-19, and some of the clinical observations have shown that the condition of some patients with COVID-19 deteriorates rapidly.^1,2,3,4^

With the increasing number of confirmed cases and the accumulating clinical data, in addition to the common clinical presentation of respiratory failure caused by COVID-19, the cardiovascular manifestations induced by this viral infection has generated considerable concern. Huang et al^5^ reported that 12% of patients with COVID-19 were diagnosed as having acute myocardial injury, manifested mainly by elevated levels of high-sensitive troponin I. From other recent data, among 138 hospitalized patients with COVID-19, 16.7% had arrhythmias and 7.2% had acute myocardial injury.^6^ However, at present, specific information characterizing whether patients with COVID-19 with underlying cardiovascular disease (CVD) who develop myocardial injury during hospitalization face greater risk and have worse in-hospital outcomes remains unknown. The present study investigated the association of underlying CVD and myocardial injury with fatal outcomes of patients with COVID-19.

## Methods

### Study Design and Participants

This single-center, retrospective, observational study was performed at the Seventh Hospital of Wuhan City, China, which is a designated hospital to treat patients with COVID-19 and supervised by the Zhongnan Hospital of Wuhan University in Wuhan, China. We retrospectively analyzed patients with COVID-19 who were diagnosed according to the interim guidance of the World Health Organization^7^ from January 23, 2020, to February 23, 2020, and who were either treated and discharged or died during hospitalization. Clinical information was collected on admission and during hospitalization by attending physicians.

This study complied with the edicts of the 1975 Declaration of Helsinki^8^ and was approved by the institutional ethics board of Zhongnan Hospital of Wuhan University and the Seventh Hospital of Wuhan City (no. 2020026). Consent was obtained from patients or patients’ next of kin.

### Data Collection

The electronic medical records of the patients were reviewed by a trained team of physicians who worked in Seventh Hospital of Wuhan City during the epidemic period. Patient data including demographics, medical history, laboratory examinations, comorbidities, complication, treatment measures (antiviral, antibiotic, corticosteroid therapies, immune glucocorticoid therapy, and respiratory support), and outcomes were collected and analyzed.

### Outcome

The end point was incidence of COVID-19–associated death. Successful treatment toward hospital discharge comprised relieved clinical symptoms, normal body temperature, significant resolution of inflammation as shown by chest radiography, and at least 2 consecutive negative results shown by real-time reverse transcription–polymerase chain reaction assay^6^ for COVID-19.

Acute respiratory distress syndrome was defined according to the Berlin Definition.^9^ Malignant arrhythmia was defined as rapid ventricular tachycardia lasting more than 30 seconds, inducing hemodynamic instability and/or ventricular fibrillation. Patients were considered to have acute myocardial injury if serum levels of troponin T (TnT) were above the 99th percentile upper reference limit.^5^

### Statistical Analysis

Categorical variables are shown as frequency rates and percentages, and continuous variables as mean (SD) and median (interquartile range [IQR]). The means for continuous variables were compared using independent group *t* tests when the data were normally distributed, otherwise, the Mann-Whitney test was used. The Pearson correlation coefficient and Spearman rank correlation coefficient were used for liner correlation analysis. Proportions for categorical variables were compared using the χ^2^ test, although the Fisher exact test was used when data were limited. Wilcoxon rank sum matched-pair tests were used to assess differences among the admission, hospitalization, and impending death. All statistical analyses were performed with SPSS, version 19.0 (IBM Corp) for Windows. A 2-sided *P* < .05 was considered statistically significant. Analysis began February 25, 2020.

## Results

### Clinical Characteristics on Admission

Data were collected in consecutive patients hospitalized with COVID-19, including 211 patients who were successfully treated and discharged and 45 patients who died. We excluded 67 discharged patients and 2 patients who died because of incomplete data, leaving 144 discharged individuals and 43 individuals who died included for final analysis. Of 187 patients, 66 (35.3%) had underlying CVD including hypertension, coronary heart disease, and cardiomyopathy, and 52 (27.8%) exhibited myocardial injury as indicated by elevated TnT levels.

On admission, none showed evidence of acute myocardial infarction, chronic liver disease, thromboembolic diseases, or rheumatism. In patients with elevated plasma TnT levels who eventually were discharged or died, the median (IQR) duration from illness onset to discharge or death was 28 (22-33) and 23.5 (18.25-34.5) days, respectively. Mortality was markedly higher in patients with elevated plasma TnT levels than in patients with normal TnT levels (31 [59.6%] vs 12 [8.9%]) (Table 1).

**Table 1.** Demographics and Clinical Characteristics of Patients With COVID-19

Characteristic	No. (%)			*P* value^a^
	Total	TnT level		
		Normal	Elevated	
No. of patients	187	135	52	NA
Male	91 (48.7)	57 (42.2)	34 (65.4)	.005
Age, mean (SD), y	58.50 (14.66)	53.53 (13.22)	71.40 (9.43)	<.001
Smoking	18 (9.6)	11 (8.1)	7 (13.5)	.27
Hospitalization, mean (SD), d	16.63 (8.12)	17.27 (7.68)	14.94 (9.03)	.08
Duration, mean (SD), d^b^	26.30 (8.96)	27.49 (8.55)	23.23 (9.35)	.003
Comorbidities				
Hypertension	61 (32.6)	28 (20.7)	33 (63.5)	<.001
CHD	21 (11.2)	4 (3.0)	17 (32.7)	<.001
Cardiomyopathy	8 (4.3)	0 (0)	8 (15.4)	<.001
Diabetes	28 (15.0)	12 (8.9)	16 (30.8)	<.001
COPD	4 (2.1)	0 (0)	4 (7.7)	.001
Malignant neoplasm	13 (7.0)	7 (5.2)	6 (11.5)	.13
Chronic kidney disease	6 (3.2)	1 (0.7)	5 (9.6)	.002
ACEI/ARB use history	19 (10.1)	8 (5.9)	11 (21.1)	.002
Complication				
ARDS	46 (24.6)	16 (11.9)	30 (57.7)	<.001
VT/VF	11 (5.9)	2 (1.5)	9 (17.3)	<.001
Acute				
Coagulopathy	42 (34.1)	17 (20.0)	25 (65.8)	<.001
Liver injury	19 (15.4)	14 (16.5)	5 (13.2)	.89
Kidney injury	18 (14.6)	4 (4.7)	14 (36.8)	<.001
Therapy				
Antivirus	166 (88.8)	120 (88.9)	46 (88.5)	.93
Antibiotic	183 (97.9)	131 (97.0)	52 (100.0)	.21
Glucocorticoid	106 (56.7)	69 (51.1)	37 (71.2)	.01
Immune globulin	21 (11.2)	14 (10.4)	7 (13.5)	.*5*
Mechanical ventilation	45 (24.1)	14 (10.4)	31 (59.6)	<.001
Clinical outcome				
Death	43 (23.0)	12 (8.9)	31 (59.6)	<.001

Abbreviations: ACEI, angiotensin-converting enzyme inhibitor; ARB, angiotensin receptor blocker; ARDS, acute respiratory distress syndrome; CHD, coronary heart disease; COPD, chronic obstructive pulmonary disease; COVID-19, coronavirus disease 2019; NA, not applicable; TnT, troponin T; VF, ventricular fibrillation; VT, ventricular tachycardia.

^a^ Statistical differences between the normal TnT and elevated TnT groups.

^b^ Duration indicates days from onset of symptoms to death or discharge.

Compared with patients with normal TnT levels (Table 1), those with elevated TnT levels were older (mean [SD] age, 71.40 [9.43] vs 53.53 [13.22]) and had a higher proportion of men (34 [65.4%] vs 57 [42.2%]). Patients with elevated TnT levels had significantly higher rates of comorbidities including hypertension (33 [63.5%] vs 28 [20.7%]), coronary heart disease (17 [32.7%] vs 4 [3.0%]), cardiomyopathy (8 [15.4%] vs 0), diabetes (16 [30.8%] vs 12 [8.9%]), chronic obstructive pulmonary disease (4 [7.7%] vs 0), and chronic kidney disease (1 [0.7%] vs 5 [9.6%]). Rates of smoking and malignant neoplasms did not differ between those with normal (11 [8.1%] vs 7 [13.5%]) and elevated TnT levels (7 [5.2%] vs 6 [11.5%]).

### Laboratory Findings on Admission

Patients with elevated TnT levels presented with significantly higher white blood cell count (median [IQR], 4640 [6170-3740] vs 7390 [4890-11 630] /μL [to convert to ×10^9 ^per liter, multiply by 0.001]) and neutrophil counts (median [IQR], 3070 [2350-4870] vs 6010 [3540-10 120] /μL [to convert to ×10^9^ per liter, multiply by 0.001]) (*P* < .001 for both) and lower lymphocyte counts (median [IQR], 840 [630-1130] vs 690 [340-1010] /μL [to convert to ×10^9^ per liter, multiply by 0.001; *P* = .01) than those with normal TnT levels (Table 2). Patients with elevated TnT levels also had significantly longer prothrombin time (median [IQR], 12.4 [12.0-13.0] vs 13.3 [12.2-15.3] seconds; *P* = .005), shorter activated partial thromboplastin time (median [IQR], 31.2 [27.5-33.2] vs 32.7 [31.0-35.8] seconds; *P* = .003), and a significant higher level of D-dimer (median [IQR], 0.29 [0.17-0.60] vs 3.85 [0.51-25.58] μg/mL [to convert to nanomoles per liter, multiply by 5.476]; *P* < .001). Hemoglobin and neutrophil counts of the 2 groups were similar.

**Table 2.** Laboratory Results Among Different Groups

Characteristic	Median (IQR)			*P* value^a^
	Total	TnT level		
		Normal	Elevated	
No. of patients	187	135	52	NA
Complete blood cell count, /μL				
White blood cell	4970 (3810-7460)	4640 (6170-3740)	7390 (4890-11 630)	<.001
Neutrophil	3700 (2410-6120)	3070 (2350-4870)	6010 (3540-10 120)	<.001
Lymphocyte	810 (560-1060)	840 (630-1130)	690 (340-1010)	.01
Coagulation profiles				
Prothrombin time, s	12.8 (12.0-14.0)	12.4 (12.0-13.0)	13.3 (12.2-15.3)	.005
APTT, s	32.0 (30.1-35.0)	32.7 (31.0-35.8)	31.2 (27.5-33.2)	.003
D-dimer, μg/mL	0.43 (0.19-2.66)	0.29 (0.17-0.60)	3.85 (0.51-25.58)	<.001
Blood lipids and electrolytes				
Cholesterol, mg/dL				
Total, mean (SD)	137.45 (34.75)	139.38 (35.14)	132.82 (33.20)	.27
Triglyceride	85.84 (62.83-123.01)	82.30 (59.29-115.04)	92.04 (69.91-159.29)	.04
HDL, mean (SD)	43.24 (10.42)	44.02 (10.81)	40.93 (8.88)	.08
LDL, mean (SD)	77.99 (25.48)	79.15 (25.87)	75.29 (23.94)	.42
Serum				
Potassium, mEq/L	3.67 (3.35-3.98)	3.67 (3.34-3.96)	3.62 (3.36-4.23)	.51
Calcium, mg/dL	8.52 (8.16-8.96)	8.60 (8.24-9.00)	8.36 (8.08-8.76)	.01
Inflammatory biomarkers				
hsCRP, mg/dL	4.04 (1.64-8.14)	3.13 (1.24-5.75)	8.55 (4.87-15.165)	<.001
Procalcitonin, ng/mL	0.08 (0.04-0.16)	0.05 (0.04-0.11)	0.21 (0.11-0.45)	<.001
Globulin, g/L	27.7 (25.8-31.0)	27.4 (25.6-29.6)	29.7 (27.0-34.6)	<.001
Other cardiac biomarkers				
Creatine kinase–MB fraction, ng/mL	1.14 (0.66-2.95)	0.81 (0.54-1.38)	3.34 (2.11-5.80)	<.001
Myoglobin, μg/L	38.5 (21.0-78.0)	27.2 (21.0-49.8)	128.7 (65.8-206.9)	<.001
NT-proBNP, pg/mL	268.4 (75.3-689.1)	141.4 (39.3-303.6)	817.4 (336.0-1944.0)	<.001
Blood gas analysis				
Pao_2_, mm Hg	83.0 (64.8-118.0)	91.0 (75.0-121.0)	64.0 (51.0-93.0)	<.001
Pao_2_/FiO_2_, mm Hg	366.7 (202.3-447.8)	390.5 (285.7-461.9)	153.3 (103.3-323.8)	<.001
Lactic acid, mm Hg	1.80 (1.40-2.25)	1.80 (1.30-2.10)	2.10 (1.40-3.10)	.004
HCO_3_, mEq/L	25.2 (22.9-27.7)	25.7 (23.8-27.9)	23.3 (20.0-27.1)	.001
Liver and renal function				
Aminotransferase, U/L				
Alanine	23.0 (14.0-35.0)	23.0 (14.0-33.0)	28.5 (16.2-39.8)	.11
Aspartate	21.0 (22.0-31.0)	29.0 (21.0-39.0)	39.5 (27.2-57.8)	<.001
Creatinine, mg/dL	0.69 (0.58-0.84)	0.63 (0.55-0.79)	0.79 (0.71-1.17)	<.001

Abbreviations: APTT, activated partial thromboplastin time; HDL, high-density lipoprotein; hsCRP, high-sensitivity C-reactive protein; IQR, interquartile range; LDL, low-density lipoprotein; NA, not applicable; NT-proBNP, N-terminal pro–brain natriuretic peptide; TnT, troponin T.

SI conversion factor: To convert aminotransferase to microkatal per liter, multiply by 0.0167; blood cell counts to ×10^9^ per liter, multiply by 0.001; calcium to millimoles per liter, multiply by 0.25; cholesterol to millimoles per liter, multiply by 0.0259; creatinine to μmol/L, multiply by 88.4; creatine kinase–MB fraction to micrograms per liter, multiply by 1; CRP to milligrams per liter, multiply by 10; D-dimer to nanomoles per liter, multiply by 5.476; HCO_3_ to millimoles per liter, multiply by 1; myoglobin to nanomoles per liter, multiply by 0.05814; NT-proBNP to ng/L, multiply by 1; triglyceride to millimoles per liter, multiply by 0.0113; potassium to millimoles per liter, multiply by 1.

^a^ Statistical differences between the normal TnT and elevated TnT groups.

Total, high-density lipoprotein, and low-density lipoprotein cholesterol levels did not differ according to TnT levels, but patients with elevated TnT levels had higher triglyceride levels (median [IQR], 92.04 [69.91-159.29] vs 82.30 [59.29-115.04] mg/dL [to convert to millimoles per liter, multiply by 0.0259]; *P = *.04). The inflammatory biomarkers, including high-sensitivity C-reactive protein (median [IQR], 8.55 [4.87-15.165] vs 3.13 [1.24-5.75] mg/dL [to convert to milligrams per liter, multiply by 10]), procalcitonin (median [IQR], 0.21 [0.11-0.45] vs 0.05 [0.04-0.11] ng/mL), and globulin (median [IQR], 29.7 [27.0-34.6] vs 27.4 [25.6-29.6] grams per liter) were significantly higher in patients with elevated TnT levels (*P* < .001 for all).

Notably, patients with normal and elevated TnT levels differed with respect to multiple indexes of organ function including the heart, liver, kidney, and lungs (Table 2). Those with elevated TnT levels had significantly higher levels of other biomarkers of cardiac injury, specifically creatine kinase–myocardial band test (median [IQR], 3.34 [2.11-5.80] vs 0.81 [0.54-1.38], ng/mL [to convert to micrograms per liter, multiply by 1]) and myoglobin (median [IQR], 128.7 [65.8-206.9] vs 27.2 [21.0-49.8] µg/L [to convert to nanomoles per liter, multiply by 0.05814]) (*P* < .001, for all) and also had higher levels of N-terminal pro–brain natriuretic peptide (NT-proBNP) (median [IQR], 817.4 (336.0-1944.0] vs 141.4 [39.3-303.6] pg/mL [to convert to nanograms per liter, multiply by 1]). Patients with elevated TnT levels had evidence of more severe respiratory dysfunction, with lower partial pressure of oxygen (Pao_2_) (median [IQR], 64.0 [51.0-93.0] vs 91.0 [75.0-121.0] mm Hg), HCO_3_ (median [IQR], 23.3 [20.0-27.1] vs 25.7 [23.8-27.9] mEq/L [to convert to to millimoles per liter, multiply by 1]), and Pao_2_/fraction of inspired oxygen (FiO_2_) (median [IQR], 153.3 [103.3-323.8] vs 390.5 [285.7-461.9] mm Hg), and higher levels of lactic acid (median [IQR], 2.10 [1.40-3.10] vs 1.80 [1.30-2.10] mm Hg) (*P* < .001, *P* < .001, *P* = .004, *P* = .001, respectively). Those with elevated TnT levels also had higher levels of creatinine (median [IQR], 0.79 [0.71-1.17] vs 0.63 [0.55-0.79] mg/dL [to convert to micromoles per liter, multiply by 88.4]) and aspartate aminotransferase (median [IQR], 39.5 [27.2-57.8] vs 29.0 [21.0-39.0] U/L [to convert to microkatal per liter, multiply by 0.0167]) (*P* < .001, both), but alanine aminotransferase did not differ between the 2 groups.

**Figure 1.**
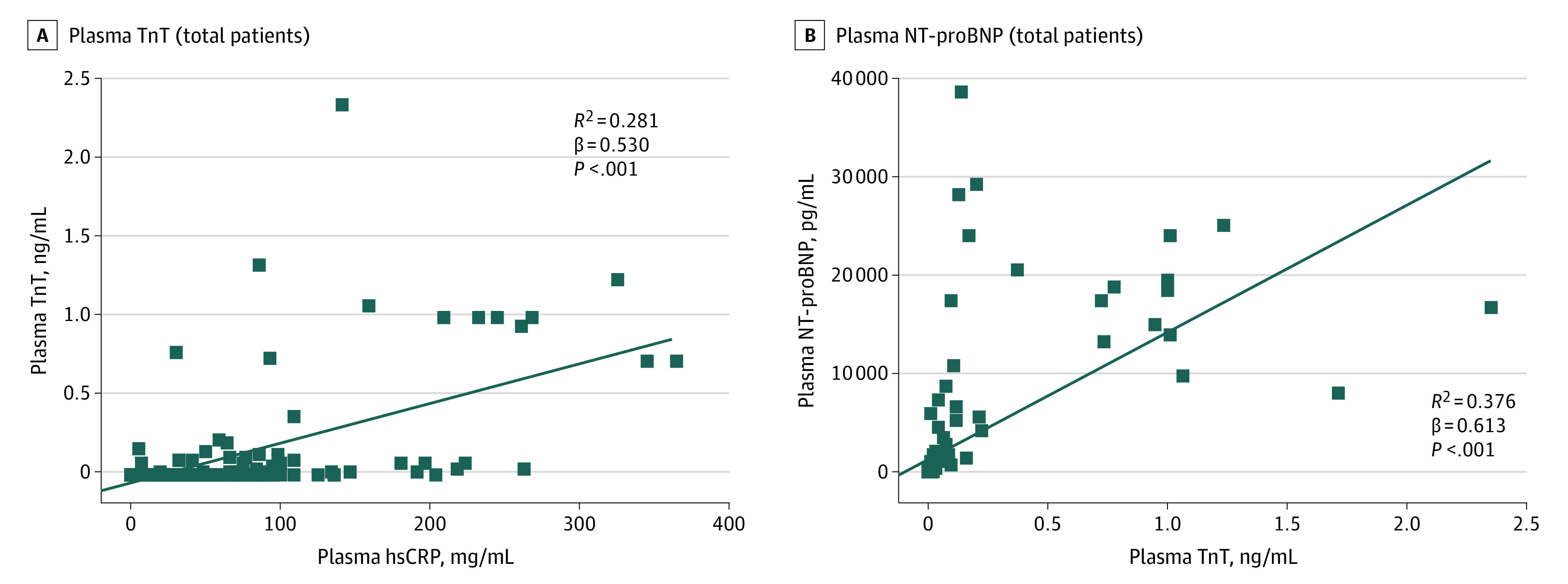
Correlation Between Plasma TnT and NT-proBNP With hsCRP Plasma troponin T (TnT), high-sensitivity C-reactive protein levels (hsCRP), and N-terminal pro–brain natriuretic peptide (NT-pro BNP) collected on admission.

Plasma TnT levels in patients with COVID-19 correlated significantly with both plasma high-sensitivity C-reactive protein levels (β = 0.530, *P* < .001) (Figure 1A) and plasma NT-proBNP levels (β = 0.613, *P* < .001) (Figure 1B).

### Comparison of Complications and Treatment During Hospitalization

Patients with underlying CVD were more likely to exhibit elevation of TnT levels (36 [54.5%]) compared with patients without CVD (16 [13.2%]). During hospitalization, patients with elevated TnT levels developed more frequent complications (Table 1), including acute respiratory distress syndrome (30 [57.7%] vs 16 [11.9%]), malignant arrhythmias (6 [11.5%] vs 7 [5.2%]) including ventricular tachycardia/ventricular fibrillation, acute coagulopathy (25 [65.8%] vs 17 [20.0%]), and acute kidney injury (14 [36.8%] vs 4 [4.7%]), compared with those with normal TnT levels. However, there was no significant differences in incidence of acute liver injury between the 2 groups. Antiviral (oseltamivir, 75 mg twice a day; ribavirin, 0.5 g twice a day; umifenovir, 0.2 g 3 times a day), antibacterial (moxifloxacin, 0.4 g every day), glucocorticoid (methylprednisolone, 40-80 mg every day), and respiratory support were the main treatment approaches for the hospitalized patients (Table 1). During hospitalization, the majority of patients underwent antiviral and antibacterial therapy, with no significant difference in such therapies between patients with normal and elevated TnT levels. However, the rates of glucocorticoid therapy and mechanical ventilation were much higher in patients with elevated TnT levels compared with those with normal TnT levels.

Long-term outpatient medications prior to admission, such as antihypertensive drugs and hypoglycemic drugs, were not discontinued. Notably, the use of angiotensin-converting enzyme inhibitors (ACEIs)/angiotensin receptor blockers (ARBs) was higher in patients with elevated TnT levels (11 [21.1%] vs 8 [5.9%]; Table 1), reflecting the higher rates of CVD. The mortality rates of patients with and without use of ACEIs/ARBs was 36.8% (7 of 19) and 21.4% (36 of 168) (*P* = .13).

### Mortality of Patients With COVID-19 With/Without CVD and With/Without Elevated TnT Levels

**Figure 2.**
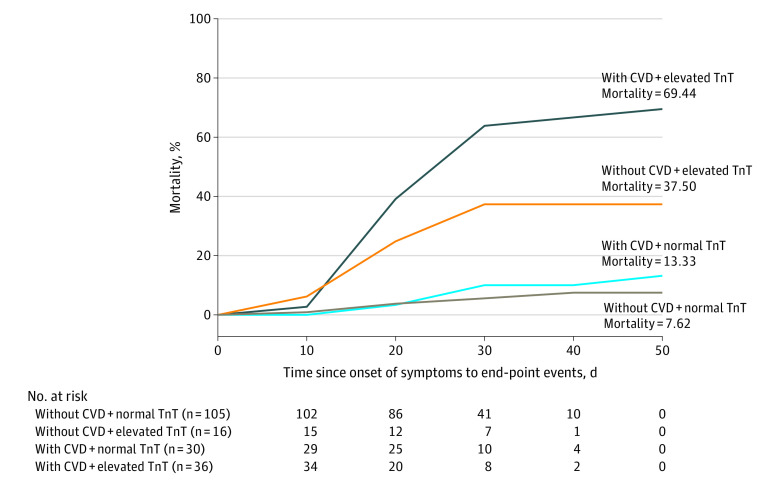
Mortality of Patients With Coronavirus Disease 2019 (COVID-19) With/Without Cardiovascular Disease (CVD) and With/Without Elevated Troponin T (TnT) Levels

Among 187 patients, 7.62% (8 of 105) with normal TnT levels without underlying CVD, 13.33% (4 of 30) with normal TnT levels with underlying CVD, 37.50% (6 of 16) with elevated TnT levels without underlying CVD, and 69.44% (25 of 36) with elevated TnT levels with underlying CVD died during hospitalization (Figure 2).

### Dynamic Changes of TnT and NT-proBNP Levels During Hospitalization

**Figure 3.**
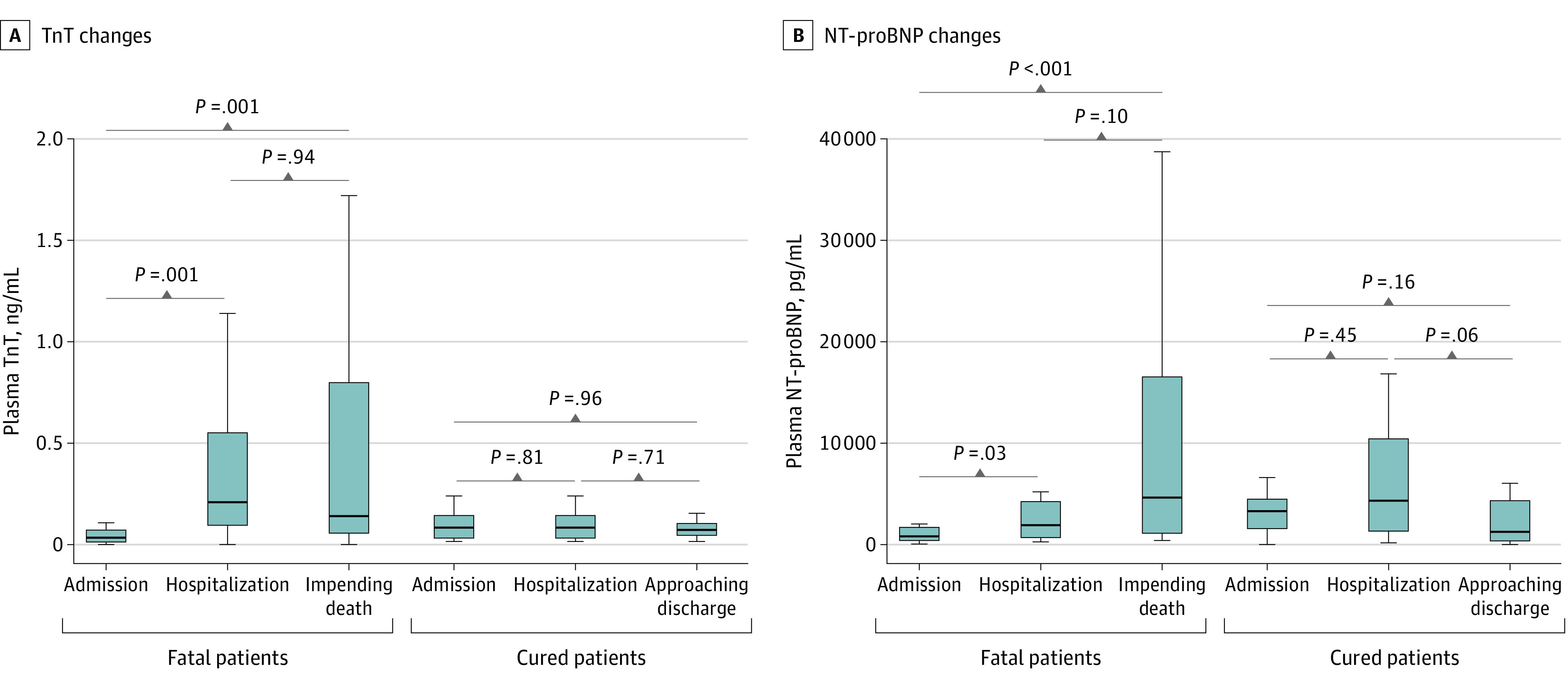
Dynamic Changes of TnT and NT-proBNP During Hospitalization The horizontal lines represent the median value in each group. NT-proBNP indicates N-terminal pro–brain natriuretic peptide; TnT, troponin T.

Figure 3 shows the dynamic escalation of TnT and NT-proBNP levels for patients who died and those who were successfully treated and discharged. Both TnT and NT-proBNP levels increased significantly during the course of hospitalization in those who ultimately died, but no such dynamic changes of TnT or NT-proBNP levels were evident in survivors.

## Discussion

### Association of Myocardial Injury With Prognosis

This report provides detailed cardiovascular information of the association between underlying CVD, myocardial injury, and fatal outcomes of patients with COVID-19. The Chinese Center for Disease Control and Prevention recently published the largest case series to date of COVID-19 in mainland China; the overall case fatality rate was 2.3% (1023 deaths among 44 672 confirmed cases), but the mortality reached 10.5% in patients with underlying CVD.^10^

In the current study, among 187 patients with COVID-19, 52 (27.8%) exhibited myocardial injury as demonstrated by elevation of TnT levels, and the mortality was markedly higher in patients with elevated TnT levels than in patients with normal TnT levels (59.6% vs 8.9%). The median (IQR) duration from illness onset to death was 23.23 (8-41) days in the group with elevated TnT levels. Patients with underlying CVD and escalation of TnT levels had the highest mortality (69.44%) and the shortest survival term. However, patients with underlying CVD but with normal TnT levels during the course of disease experienced a more favorable prognosis, compared with patients with elevated TnT levels but without underlying CVD (mortality, 13.3% vs 37.5%). The dynamic escalation of NT-proBNP and increased incidence of malignant arrhythmias during the course of disease in patients with elevated TnT levels is evidence that myocardial injury played a greater role in the fatal outcome of COVID-19 than the presence of underlying CVD itself.

NT-proBNP elevation and malignant arrhythmias were significantly more common in patients with elevated TnT levels, and NT-proBNP was significantly correlated with TnT levels (Figure 1). This suggests that those with myocardial injury were more likely to experience impairment in cardiac function.

### Potential Mechanism Underlying Myocardial Injury

The current study demonstrates that patients with underlying CVD and other comorbid conditions are more prone to experience myocardial injury during the course of COVID-19. For patients with underlying CVD, including hypertension, coronary heart disease, and cardiomyopathy, viral illness can further damage myocardial cells through several mechanisms including direct damage by the virus, systemic inflammatory responses, destabilized coronary plaque, and aggravated hypoxia. Therefore, patients with CVD are more likely to experience myocardial injury after COVID-19 infection and higher risk of death. However, it is also notable that the 16% of patients with underlying CVD but with normal TnT levels had a relatively favorable outcome in this study. These data suggest that myocardial biomarkers should be evaluated in patients with CVD who develop COVID-19 for risk stratification and possible early and more aggressive intervention.

Although the exact pathophysiological mechanism underlying myocardial injury caused by COVID-19 is not fully understood, a previous report showed that in 35% of the patients with severe acute respiratory syndrome coronavirus (SARS-CoV) infection, the SARS-CoV genome was positively detected in the heart. This raises the possibility of direct damage of cardiomyocytes by the virus.^11^ SARS-CoV-2 may share the same mechanism with SARS-COV because the 2 viruses are highly homologous in genome.^12,13^ In the current study, plasma TnT levels were significantly positively linear correlated with plasma high-sensitivity C-reactive protein levels (Figure 2), indicating that myocardial injury may be closely associated with inflammatory pathogenesis during the progress of disease. Viral particles spread through respiratory mucosa and simultaneously infect other cells, which could precipitate a cytokine storm and a series of immune responses. Huang et al^5^ highlighted that in patients with COVID-19, the imbalance of T helper 1 and T helper 2 responses resulted in a cytokine storm, which may contribute to myocardial injury. The release of inflammatory cytokines after infection may cause reduction in coronary blood flow, decreases in oxygen supply, destabilization of coronary plaque, and microthrombogenesis.

### Consideration of Prevention and Treatment for Myocardial Injury

Unfortunately, until now, no specific antiviral drugs or vaccines have been recommended for COVID-19 except for symptomatic supportive treatment and intervention. As patients with underlying CVD are more likely to develop more severe adverse outcomes when myocardial injury occurs after COVID-19 infection and face higher risk of death, it may be reasonable to triage patients with COVID-19 according to the presence of underlying CVD and evidence of myocardial injury for prioritized treatment and even more aggressive treatment strategies. Other cardiac biomarkers such as NT-proBNP and electrocardiograms should be closely monitored for early warning and intervention.

There remains controversy concerning the use of ACEI/ARB for COVID-19. In this study, with a limited number of patients, the mortality of those treated with or without use of ACEI/ARB did not show a significant difference in outcome. Concerns about ACEI/ARB have been raised since angiotensin-converting enzyme 2 (ACE2) is a potential target for COVID-19 infection, and the increased ACE2 expression induced by ACEI or ARB would aggravate lung injury of patients with COVID-19. However, a previous study^14^ showed a beneficial effect of ACEI/ARB in patients admitted with viral pneumonia, as it significantly reduced the pulmonary inflammatory response and cytokine release caused by virus infection. The beneficial effect of ACEI/ARB may be related to a compensatory increase in ACE2.^15^ However, the evidence regarding the use of ACEI/ARB in patients with COVID-19 infection is still emerging, and larger clinical studies are required. At present, for patients with COVID-19 who previously used ACEI/ARB, the use of these drugs may not need to be discontinued based on current data.

### Limitations

Our study has several limitations. First, only 187 patients with confirmed COVID-19 were included, and a larger cohort study is needed to verify our conclusions. Second, as a retrospective study, some other specific information regarding cardiovascular complications and inflammation such as echocardiography and interleukin 6 were not presented in the study because the data were incomplete owing to the limited conditions in the isolation ward and the urgency of containing the COVID-19 epidemic. Third, the data in this study permit a preliminary assessment of the clinical course and outcomes of patients with COVID-19. The causes of death may involve multiple organ dysfunction in most cases, and it is difficult to differentiate the myocardial injury as the main and direct cause in an individual case. Long-term observation and prospective study design on the effectiveness of treatments specific for the myocardial injury are needed.

## Conclusions

Myocardial injury has a significant association with fatal outcomes of COVID-19, while the prognosis of patients with underlying CVD but without myocardial injury appears relatively favorable. Myocardial injury is associated with impairment of cardiac function and ventricular tachyarrhythmias. Inflammation may be associated with myocardial injury. Aggressive treatment may be considered for the patients with myocardial injury.
